# A Case of Strangulated Femoral Hernia Successfully Punctured, with Subsequent Spontaneous Reduction

**Published:** 1873-08

**Authors:** J. Suydam Knox

**Affiliations:** Chicago


ARTICLE III.—*A Case of Strangulated Femoral Hernia Successfully Punctured, with Subsequent Spontaneous Reduction.* By J. SUYDAM KNOX, M.D., Chicago.

Maria Van D——, a feeble colored woman, about 73 years of age, has suffered from femoral hernia of the right side, ever since her last confinement, nearly thirty years ago. On many occasions has it become strangulated, and been reduced by taxis. In the evening of the 20th of August, 1872, I was called to see her. The hernia had been strangulated over twenty-four hours, presenting a tumor over three inches in diameter and nearly five inches long. The patient was much prostrated and suffering acutely. Chloroform was twice administered; first, to reduce by taxis, and when that failed, a second time, for the purpose of operating; but in each case its use had to be abandoned on account of the dangerous symptoms it produced. Under such circumstances the hernial tumor was punctured. The hypodermic syringe was used. After unscrewing the cap and removing the piston, the needle was pushed its full length into the apex of the tumor, and immediately a bloody serum, of decidedly fœcal odor, commenced to flow, without any escape of gas. After eight and a half ounces had thus escaped, the hernia reduced itself spontaneously. A third of a grain of morphia was then injected subcutaneously.

The patient was visited on the two following days. There was no increase of temperature, and no abdominal tenderness. The slight puncture of the intestine seemed to provoke no peritoneal inflammation, nor was there any apparent leakage from the gut after reduction was accomplished. In fact, recovery was complete as soon as the hypnotic effect of the morphia had passed off.

I report this case simply as being a suggestive one. Every one who has operated for strangulated hernia, knows the care and skill required; the danger of injury to the gut, or close-lying artery; the occasional result of peritonitis, or artificial anus; and in addition, the small percentage of risk which always accompanies wounds of large cavities and the use of anæsthetics.

Every surgeon also is familiar with the general success of paracentesis of the thorax, abdomen and scrotum, and with the fact that tapping in hydro-pericarditis and puncture of the intestines in excessive tympanitis, have been performed with impunity.

Why then should not puncture be generally adopted for strangulated hernia?

My own impression is, that, as in this case, the contents of the strangulated gut are always largely transuded serum, the result of compression of the veins at the hernial ring—such transudation invariably happens where the venous circulation is arrested, the arteries remaining patulous. If this transuded fluid is removed, the contents are no more than readily entered the ring and can as easily be returned.

Again, puncture can be followed by but one of the many results of the use of the knife, viz: peritonitis.

Briefly then, if the contents of the strangulated gut are fluid, and if puncture be innocent of evil consequences, it should be preferred to the use of the bistoury for the following reasons:

1. It is most readily and easily performed.
2. It is attended by far less risk of injury.
3. It requires no anæsthesia.

